# The Mouth in Backward Children (Imbecile) of the Mongolian Type

**Published:** 1889-12

**Authors:** Robert Jones


### Editorial, Etc.

---

THE MOUTH IN BACKWARD CHILDREN (IMBECILE) OF THE MONGOLIAN TYPE.—An interesting paper on this subject appears in the *London Dental Reeord* of December, 1889, as follows: "So much has been said and written about the mouth—the palate and the teeth— that it may be difficult to say anything fresh, perhaps more difficult to reconcile statements already made. We hear from one authority that a highly arched palate and contracted jaws are a sure sign of weak-mindedness; so sure, that imbecility can be diagnosed as congenital or otherwise according to their presence. Another authority regards contracted, badly-developed jaws, as an equally certain sign of a higher state of civilization—children of a "well bred aspect" having the abnormality in about seventy per cent.; those of a "coarse, low, and brutal aspect" manifested the deformity in only seven to eight per cent., whereas those children of doubtful aspect occupied an intermediate position between the other two as regards the frequency of the deformity. The comparative disuse of the organs of mastication among civilized races who cook and soften their food before masticating, is probably confirmed by a statement made that irregularities of the teeth and contracted jaws are rare among savage races. It has at any rate become very generally acknowledged that a highly-arched palate is a common deformity in imbeciles, and the importance of the teeth as organs of mastication, with their necessity for healthy digestion, has led to certain observations which I shall shortly relate. From an examination of a large number of mouths in imbecile children, I am very strongly convinced that vaulted arches are not so common as has been supposed, and that these high palates occur mostly in two classes of imbeciles, viz., the micro-cephalic and the Mongolian type. As it is much more common in the latter, I shall refer only to this class. It is well known that there are among imbeciles as a genus several well-defined and distinct classes; no class is more clearly defined and more distinct than the class described by one authority in his ethnological classification as the Mongol or Kalmuck type. This class, composed of individuals having a very close resemblance to one another, is not a very large one; the species constitute about four or five per cent. of the total number of imbecile children. They are generally short in stature—adult males rarely measure over five feet; females rarely over fifty-five inches in height—they have a squat figure, and are either of a very light or dark complexion; the head is small and round, the measurements from the root of the nose to the occipital protuberance, and from ear to ear, being nearly equal. The hair is never curly, it is straight, lank and thin, or straight and coarse, sometimes absent, at other times devoid of pigment; the faces are round, length and breadth being nearly equal, the nose is usually flat over the bridge, and upturned and sharp as if bitten off; the eyebrows tend to run outwards and slightly upwards, the eyelids more so, the outer canthus and the opening between the eyelids having a very distinct onward and upward tendency—hence the Chinese, Tartar, or Mongolian features—the eyebrows may have a distinctly arched direction, but the outer canthus has an almost invariable upward direction. The space between the eyes appears wider than normal, owing to the flattened condition of the bridge of the nose. The face is rough, the skin being generally coarse and loose; the tongue is usually deeply transversely fissured; their hands and feet are broad and short, and their joints are very supple, their usual and favorite position at ease being that of squatting, tailor-fashion, on their crossed legs, again like the Chinese. Their mental no less than their physical features are characteristic, the habits being lethargic, dull and reserved in company; they are very observant, not easily roused to enthusiasm by others, although vere playful and original when not watched; they are very apt mimics of muscular movements, fond of music and color, they can be educated with advantage up to a certain point and have a great desire when it pleases them to be useful to others. The smaller children of this type will turn over and over again the gaudy pages of a colored picture book, in appreciation of varied tints. They are often near-sighted. Their articulation is defective; they can rarely pronounce the sibilants, but improve by training; they are usually short-lived, but rarely do any of this class suffer from epilepsy. They are generally the youngest in large families, or there is much disparity in the parents' ages. It is very desirable to know this type in order to give a satisfactory prognosis to anxious parents.

Now with regard to the teeth and palate. As stated above, I am strongly of opinion that too much stress is laid upon a highly arched palate as a concomitant deformity in the weak-minded child. I am far from saying that this congenital irregularity does not occur, but it occurs more frequently in the Mongolian, which type forms but a small percentage of imbeciles. Whether this irregularity be inherited or not, it is difficult to state; observation has proved that variations in the position of the teeth, at first sight seeming accidental, are often transmitted from parent to child, so is also the congenital absence of certain teeth; still, in some cases, it is doubtless that the high vault, mal-position of the teeth, together with irregularity of the maxillæ, may be due to development causes. Considering that the relation between dermal coverings and the teeth is a close one, as has been pointed out by Darwin in his reference to certain mammals which being most aberrant in their dermal coverings are also remarkable for the deficiency or redundancy in the number of their teeth, it is not surprising that we should find these organs and the palate peculiarly affected in Mongolian imbeciles in whom a definite and peculiar condition of the skin and hair has been found to co-exist. It is doubtless needless to refer to the fact that both the skin and its appendages with the teeth are similarly developed from the same epiblastic layer of the blastoderm, and what affects the one during intra-uterine life is likely probably to affect the other under similar conditions. The variations met with in these cases may conveniently be referred to under three headings: the *teeth*, *maxillæ*, and *palate*.

(1). *The Position of the Teeth.*—In no case was there marked prognathism, the front teeth of the upper jaw did not markedly project forward, neither was there a marked retreating backwards, but the lower incisors rarely met the upper in exactly a straight line; the most usual is for the lower jaw to be slightly more prominent than the upper, the molar teeth appear to be fairly normal, and the ramus is not unduly rectangular, perhaps the most frequent cause of projecting upper teeth. The lower incisors also appeared fairly uniform as regards enamel, although the enamel of the teeth in the lower jaw was much more frequently coated with tartar, due to the accumulation of saliva in the mouth, owing to the sluggish movements of mastication and deglutition in all children of feeble intellect, a peculiarity not by any means confined to the Mongolian type. The lower front teeth, as well as the upper, are not very regular, and it is well known that any abnormality in the position of the teeth has not the same chance of correction in the imbecile that it has in the healthy child—owing to the nerve force controlling the even and symmetrical pressure of the lips and tongue upon the teeth being impaired—the tendency to correction is, therefore, absent or diminished.

(2.) *The Maxillæ.*—The most frequent condition mentioned above, viz., the prominence of the lower line of incisors, may be referred either to a smaller alveolar ridge of the upper maxilla—possibly hereditary—or to a departure of the lower jaw from its normal form. It is unusual to find in these cases any marked deviation either in size or shape of the lower jaw, although an abnormal development (as when the teeth do not meet, being separated by a gap) in some cases must exist. In these, owing to the obliquity of the ramus of the lower jaw the molars alone meet, and that portion of the jaw in front is greatly increased compared to the portion bearing the molar teeth. In the upper jaw the contraction of the alveolus at the level of the bicuspids gives rise to the palatal deformity or irregularity which is the most constant feature in these cases, and which, unfortunately, cannot be remedied; in one child the highest point of the vault measured over 1¼ in. from the level of the crown of the teeth, and must interfere considerably with voice-production.

(3.) *Palate.*—The V-shaped or wedge-shaped mouth is the most commonly met; in it the whole of the teeth of one or both jaws may be involved, and instead of the elliptical arrangement, the teeth occupy two converging lines which meet at an angle in the anterior part of the jaw, being frequently accompanied by a high and vaulted palate. The position of the teeth on the two sides is generally pretty symmetrical, but the deformity is usually confined to the upper jaw, there being an appearance of contraction at the line of the bicuspid teeth, giving the suggestion that the jaw had been pinched at this level and the palate directed upwards. As the contraction of the jaw is said not to be manifest until the eruption of the permanent teeth, and the molar teeth generally diverge from this point of contraction at the bicuspid line, it appears that unless the front portion of the jaw (which undergoes no material alteration in form after birth) has received during intra-uterine life its imprint for posterior divergence, the newly added portion at the back of its alveolar border will form an angle with it, and this angle corresponding with the contraction of the V-shaped palate becomes the deformity (either accidental or hereditary) which is at present impossible to remedy. Other causes of contracted jaws than those occurring during intra-uterine life, such as premature extraction of temporary teeth, &c., need not here be discussed. In many of these children the enamel of the teeth is irregular, easily broken down, pitted and grooved, and it would be interesting to find out further relations between homologane structures developed from the epiblastic layer, such as the skin and appendages, the teeth and the nervous system.—*Robert Jones.*

